# Elevation in fibroblast growth factor 23 and its value for identifying subclinical atherosclerosis in first-degree relatives of patients with diabetes

**DOI:** 10.1038/srep34696

**Published:** 2016-10-04

**Authors:** Xiang Hu, Xiaojing Ma, Yuqi Luo, Yiting Xu, Qin Xiong, Xiaoping Pan, Yuqian Bao, Weiping Jia

**Affiliations:** 1 Department of Endocrinology and Metabolism, Shanghai Jiao Tong University Affiliated Sixth People’s Hospital; Shanghai Clinical Center for Diabetes; Shanghai Key Clinical Center for Metabolic Disease; Shanghai Diabetes Institute; Shanghai Key Laboratory of Diabetes Mellitus, Shanghai 200233, China

## Abstract

Accumulating evidence supported an association between diabetes and fibroblast growth factor 23 (FGF23). The goal of the present study was to explore alteration in serum FGF23 levels and to assess its value for identifying subclinical atherosclerosis in normoglycemic individuals with a first-degree family history of diabetes (FHD). The study enrolled 312 subjects with a first-degree FHD and 1407 subjects without an FHD. Serum FGF23 levels were detected by a sandwich enzyme-linked immunosorbent assay. Serum FGF23 levels were much higher in subjects with a first-degree FHD than in those without an FHD (*P* = 0.006). A first-degree FHD was positively associated with serum FGF23 levels, independent of C-IMT and cardiovascular factors (both *P* < 0.05). In subjects with a first-degree FHD, only those with serum FGF23 levels in the upper quartile were more likely to have an increased C-IMT (odds ratio = 2.263, *P* < 0.05). As conclusions, a first**-**degree FHD contributes to the increased serum FGF23 levels independently. Subjects with a first-degree FHD need higher serum FGF23 levels to indicate subclinical atherosclerosis. The influence of a first-degree FHD on serum FGF23 levels should be considered to avoid overestimating the risk of cardiovascular disease in normoglycemic individuals with a first-degree FHD.

Type 2 diabetes mellitus (T2DM) is an etiologically heterogeneous disease, and its pathogenesis is strongly correlated to both genetic and environmental factors, as well as their interactions^1^. On the basis of genetic susceptibility, the prevalence of T2DM has increased rapidly with changes in environment and lifestyle, and then is propagated by genetic susceptibility. The lifetime risk of diabetes is 40% for first-degree relatives of patients with diabetes who have one parent with T2DM, and this risk is nearly 70% if both parents have T2DM^2^. A family history of diabetes (FHD) also was proposed as a significant cardiovascular risk factor by the Multi-Ethnic Study of Atherosclerosis^3^. Endothelial dysfunction predisposes first-degree relatives of patients with diabetes to developing atherosclerosis^4^. The normoglycemic individuals with a first-degree FHD are preferable for studies investigating metabolic abnormalities in the early stage of disease development, because they share the same genetic background as diabetes patients and are known to experience metabolic alterations preceding the onset of hyperglycemia^5^.

Fibroblast growth factor 23 (FGF23) belongs to the subfamily of endocrine fibroblast growth factors, and its physiological role is to maintain the balance of mineral metabolism^6^. FGF23 is believed to be a bone-derived hormone, and primary defects in bone mineralization and turnover affect FGF23 production^6^. Additionally, 1,25-dihydroxyvitamin D, kidney, fat mass, and adipocytokines are suggested to be systemic regulators of FGF23^6^. Recently, a basic research study revealed that insulin action contributes to FGF23 production^7^, as evidenced by elevated levels of circulating FGF23 in the individuals with T2DM in most clinical studies^8^,^9^,^10^,^11^. However, some studies with small sample sizes have failed to find an association between the presence of diabetes and circulating FGF23 levels^12^,^13^. Furthermore, FGF23 is involved in the onset and progression of atherosclerosis via its effects on endothelial cell function^14^ and has been proposed as a predictor of cardiovascular disease (CVD) risk by prospective studies^15^.

Given the inconsistent findings regarding serum FGF23 levels in diabetes patients and the lack of data for serum FGF23 levels in the first-degree relatives of patients with diabetes, the present study investigated alteration in serum FGF23 levels in normoglycemic individuals with a first-degree FHD.

In addition, the carotid intima-media thickness (C-IMT) assessed by ultrasonography, which visualize the pathological characteristics of the carotid wall and lumen surfaces, is believed to be a quantitative and reproducible index to reflect the severity of systemic atherosclerosis^16^,^17^. As a non-invasive, reliable, convenient, and economical tool, carotid ultrasonography was widely used in clinical practice and epidemiological investigations^18^,^19^. Similarly, in the present study, C-IMT was applied to detection of subclinical atherosclerosis^20^ in order to evaluate whether serum FGF23 levels can be used to identify subclinical atherosclerosis in the first-degree relatives of patients with diabetes.

## Results

### Clinical characteristics of the study participants

The total study population of 1719 participants (age range: 20–78 years, median 52.82 [46.25–57.73] years) included 312 subjects with a first-degree FHD and 1407 subjects without an FHD. The proportion of women and levels of fasting plasma glucose level (FPG), glycated hemoglobin (HbA1c), fasting serum insulin (FINS), homeostasis model assessment for insulin resistance (HOMA-IR), total cholesterol (TC), and low-density lipoprotein cholesterol (LDL-c) were greater among subjects with a first-degree FHD than those without an FHD (all *P* < 0.05). No differences in the other variables were observed between the groups (all *P* > 0.05; Table 1).

### Comparison of serum FGF23 levels

**Figure 1:**
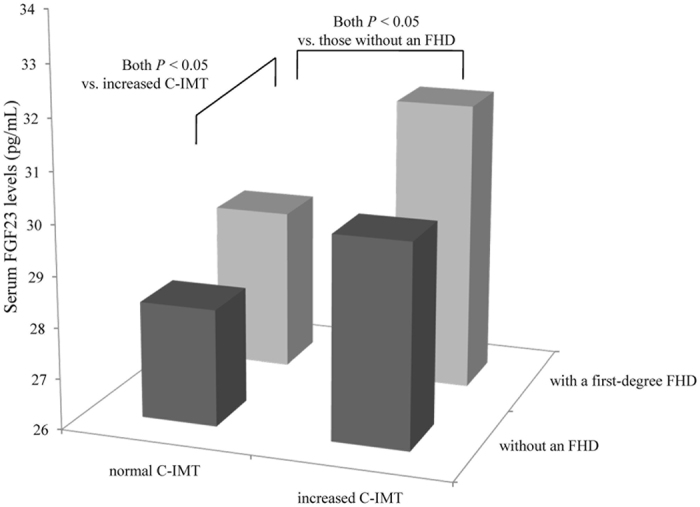
Comparison of serum FGF23 levels. Subjects with a first-degree FHD have higher serum FGF23 levels than those without an FHD both in subgroup of normal and increased C-IMT (both *P* < 0.05). Subjects with an increased C-IMT have higher serum FGF23 levels than those with a normal C-IMT whether in presence or absence of a first-degree FHD (both *P* < 0.05).

Serum FGF23 levels were much higher in subjects with a first-degree FHD than in those without an FHD (29.20 [25.00–35.99] pg/mL versus 28.30 [22.90–35.00] pg/mL, *P* = 0.006). The study population was divided into four subgroups on the basis of having a first-degree FHD and the measured C-IMT. The results showed whether in the presence or absence of an increased C-IMT, subjects with a first-degree FHD exhibited higher levels of serum FGF23 than those without an FHD (both *P* < 0.05). Additionally, among both participants with and without an FHD, serum FGF23 levels were much higher in individuals with an increased C-IMT (both *P* < 0.05; Fig. 1). Furthermore, serum FGF23 levels did not differ between the subjects with a first-degree FHD but without an increased C-IMT and the subjects with an increased C-IMT instead of a first-degree FHD (29.20 [24.20–35.00] pg/mL versus 30.00 [24.20–35.93] pg/mL; *P* = 0.977).

### Relationship between a first-degree FHD and the serum FGF23 levels

Logistic regression analysis revealed an independent and positive relationship between a first-degree FHD and the serum FGF23 levels (odds ratio [OR] = 1.023, *P* = 0.001). After adjustment for gender, age, and estimated glomerular filtration rate (eGFR), the positive association between the first-degree FHD and the serum FGF23 levels remained significantly (OR = 1.025, *P* = 0.001). Furthermore, this relationship remained significant after additional adjustment for C-IMT and cardiovascular risk factors (OR = 1.024, *P* = 0.001 and OR = 1.022, *P* = 0.003, respectively; Table 2).

### Logistic regression analysis of the presence of an increased C-IMT

Defining the presence of an increased C-IMT as a dependent variable, logistic regression analysis showed that the OR for an increased C-IMT increased with increasing serum FGF23 levels both in subjects with and without an FHD (both *P* < 0.05). The FGF23 quartiles were defined according to the median and interquartile values of serum FGF23 levels for the entire study population, as follows: quartile 1: ≤ 23.80 pg/mL; quartile 2: 23.81–29.19 pg/mL; quartile 3: 29.20–34.99 pg/mL; and quartile 4: ≥ 35.00 pg/mL. In those without an FHD, individuals with serum FGF23 levels in quartiles 3 and 4 were more likely to have an increased C-IMT (OR = 1.415 and 1.439, respectively, both *P* < 0.05). Among subjects with a first-degree FHD, only those with serum FGF23 levels in quartile 4 showed a significantly increased OR for an increased C-IMT (OR = 2.263, *P* < 0.05; Table 3).

## Discussion

In the present study, individuals with a first-degree FHD exhibited elevated serum FGF23 levels no matter in the presence or absence of an increased C-IMT, and a first-degree FHD was positively associated with the serum FGF23 levels independent of the C-IMT and other cardiovascular risk factors. For individuals with a first-degree FHD, higher serum FGF23 levels were necessary to indicate the presence of an increased C-IMT.

A previous multicenter study in the USA^8^ involving 3756 participants with mild to moderate chronic kidney disease (CKD) reported that diabetes patients had higher serum FGF23 levels and experienced an earlier onset of FGF23 excess. After adjustment for demographic, clinical, and laboratory variables, diabetes remained a significant factor associated with serum FGF23 levels in their study. Their findings also held true for study participants with an eGFR >60 mL/min/1.73 m^2^. Another study conducted in population with CKD stages 2–4 provided evidence for a positive association between the presence of diabetes and serum FGF23 levels^9^. Similarly, Schoppet *et al.*^10^ reported the association of diabetes with elevated serum FGF23 levels in healthy older men (>60 years). A community-based study proposed that an elevated FGF23 concentration is a subclinical marker of metabolic perturbations (diabetes, dyslipidemia, and obesity) in individuals with normal kidney function^11^. Therefore, the population included in the present study was restricted to those with normal glucose tolerance to eliminate the effects of hyperglycemia.

Winther *et al.*^12^ performed a euglycemic-hyperinsulinemic clamp in a small study population consisting of obese individuals with T2DM, glucose-tolerant obese healthy individuals, and lean individuals. Only in diabetes subjects did they observe a significant increase in serum FGF23 levels after clamp, which was correlated with the increase in insulin levels. Mirza *et al.*^21^ found that a 1-standard deviation increase in log FGF23 was associated with 8–12% increases in insulin levels and HOMA index values in two independent cohorts of elderly individuals. These findings suggested that hyperinsulinemia and insulin resistance might contribute to the increase in serum FGF23 levels in diabetes population.

The first-degree relatives of patients with diabetes are considered to have an increased risk of diabetes, and they often exhibit hyperinsulinemia even in absence of obesity^5^. Stadler *et al.*^22^ conducted 75 g oral glucose tolerance test and hyperinsulinaemic–isoglycaemic clamps on 706 participants without diabetes. Their findings indicated the presence of insulin resistance and β cell dysfunction in the individuals with a first-degree FHD. Another prospective cohort study^23^ of non-diabetes individuals with a first-degree FHD also demonstrated that, genetically predisposed for diabetes, individuals with deteriorating glucose tolerance exhibited insulin resistance and β cell dysfunction. Contrary to the great amount of evidence supporting the impaired insulin activity in the individuals with a first-degree FHD, no previous studies have reported the variation in serum FGF23 levels in this population. The present study revealed a significant increase in serum FGF23 levels in normoglycemic individuals with a first-degree FHD, accompanied by increases in serum insulin levels and HOMA-IR values, and these findings are consistent with those of most studies of diabetes populations. Based on these results, the heritability of diabetes could be responsible for the elevation in serum FGF23 levels, even when blood glucose levels are in the normal range. The abnormality of insulin action may be an explanatory factor for the alteration in serum FGF23 levels in the first-degree relatives of patients with diabetes.

Clinical studies have revealed that endothelium-dependent vasodilation decreases by 38%^4^, whereas the C-IMT increases by 18% in first-degree relatives of patients with diabetes^24^. Moreover, first-degree relatives of patients with diabetes were found to be more likely to develop atherosclerotic CVD^25^. Therefore, early assessment of CVD risk is particularly important for individuals with a first-degree FHD to prevent the onset and progression of CVD. A prospective study proposed that the serum FGF23 levels can predict major cardiovascular events in the community population, even in consideration of the established cardiovascular risk factors, mineral metabolism abnormalities, and subclinical cardiovascular damage^15^. An increased C-IMT represents an intermediate phenotype for early-stage atherosclerosis. Due to its association with various cardiovascular risk factors and its predictive value for CVD-related death, the C-IMT serves as a reliable index applied to detecting and monitoring atherosclerosis clinically^20^. By using the C-IMT to represent subclinical atherosclerosis, as well as evaluate the CVD risk, the present study found that an elevated serum FGF23 levels could indicate the presence of subclinical atherosclerosis in normoglycemic individuals with a first-degree FHD.

Notably, the relationship between the presence of diabetes and altered serum FGF23 levels was reported to be independent of cardiovascular risk factors in previous studies^9^. Consistently, the present study found that first-degree relatives of patients with diabetes exhibited higher levels of serum FGF23 at an equivalent degree of subclinical atherosclerosis as assessed by the C-IMT. The association of a first-degree FHD with an elevated serum FGF23 levels was independent of the C-IMT and other cardiovascular factors. For individuals with a first-degree FHD, additionally, the serum FGF23 levels increased even in absence of an increased C-IMT, reaching the same levels observed in participants with an increased C-IMT and without an FHD. Compared to those without an FHD, participants with a first-degree FHD need higher levels of serum FGF23 to indicate the subclinical atherosclerosis. Thus, the influence of a first-degree FHD on the serum FGF23 levels should be taken into consideration in the clinical setting. If not, the use of serum FGF23 as a biomarker in first-degree relatives of patients with diabetes may result in overestimation of the CVD risk.

Although the relationship between the presence of a first-degree FHD and serum FGF23 levels was demonstrated in the present study, the potential mechanism for the findings remains unclear. Insulin activation induces stimulation of Akt/PKB and serum- and glucocorticoid-inducible kinase isoforms, further resulting in the inhibition of glycogen synthase kinase (GSK)-3. Experiments in transgenic mice revealed that GSK3 was involved in the regulation of FGF23 release via the promotion of sympathetic nervous system activity^7^. Therefore, it is possible that serum FGF23 levels increase upon alteration of insulin activity in individuals with a first-degree FHD. Moreover, human studies on diabetes population provided evidence supporting the contribution of insulin resistance to the alteration in microarchitecture, osteoblast differentiation, and bone turnover, which consequently results in the poorer quality of the skeleton^26^. Although the current animal models of T2DM are limited by their ability to recapitulate the impact of diabetes on bone metabolism, the data available are largely consistent with those obtained in human studies^27^, suggesting that insulin-induced bone impairment may play a role in the elevated serum FGF23 levels in the first-degree relatives of patients with diabetes.

The cross-sectional design of this study limited the ability to assess the directionality of the association between elevated serum FGF23 levels and an increased C-IMT. A further prospective study is needed to evaluate the predictive value of serum FGF23 levels for the CVD risk in first-degree relatives of patients.

In conclusions, a first-degree FHD contributes to an increase in serum FGF23 levels, independent of the presence of subclinical atherosclerosis and cardiovascular factors. In individuals with a first-degree FHD, serum FGF23 levels need to be higher to indicate the presence of subclinical atherosclerosis. For use of serum FGF23 levels in the clinical evaluation of first-degree relatives of patients with diabetes, the influence of a first-degree FHD should be considered to avoid overestimation of the CVD risk in this population.

## Methods

### Subjects

The study population comprised a subgroup of participants with normal kidney function (eGFR ≥ 60 mL/min/1.73 m^2^) of the Shanghai Obesity Study^28^. Based on the 1999 World Health Organization criteria^29^, individuals displaying impaired glucose regulation or diabetes were excluded. Individuals with the following conditions also were excluded: established cardiovascular and cerebrovascular disease, carotid plaque, hepatic dysfunction, hyperthyroidism or hypothyroidism, hypercalcemia, acute infection, tumors, psychiatric disease, current lipid-lowering therapy, and current replacement therapy with systemic corticosteroids or thyroxine. The participants provided complete clinical data and finished a standardized questionnaire to gather information on their disease history, medication usage, family history, and smoking status. A first-degree FHD was defined as having one or more first-degree relatives of patients with diabetes (parent, sibling, or offspring)^30^.

The study was carried out in accordance with the Declaration of Helsinki and approved by the Ethics Committee of Shanghai Jiao Tong University Affiliated Sixth People’s Hospital. All participants provided written informed consent prior to participation to the study.

### Anthropometric and biochemical assessments

All participants were examined after an overnight fast lasting at least 10 hours. Anthropometric assessments included height, weight, waist circumference (W), and resting blood pressure. Body mass index (BMI) was calculated as follows: BMI = weight (kg)/height^2^ (m^2^). Biochemical variables were detected according to methods previously reported^28^, including: FPG, 2-hour plasma glucose (2 hPG; determined after a 75 g oral glucose tolerance test), HbA1c, FINS, TC, triglycerides (TG), high-density lipoprotein cholesterol (HDL-c), LDL-c, C-reactive protein (CRP), serum creatinine (Scr), and serum calcium (Ca) levels. A Kainos sandwich enzyme-linked immunosorbent assay kit (Kainos Laboratories Inc., Tokyo, Japan) was used to detect serum FGF23 levels. The intra- and inter-assay coefficients of variations were 5.6% and 8.2%, respectively. Insulin resistance was assessed using the insulin resistance index (HOMA-IR), calculated as previously described^28^. The eGFR was calculated according to the Chronic Kidney Disease Epidemiology Collaboration equation^31^: eGFR = [141 × min (Scr (mg/dL)/k, 1)^α^ × max (Scr/k, 1)^−1.209^ × 0.993^age^ × 1.018 (if women)]; where k is 0.7 for women and 0.9 for men, and α is −0.329 for women and −0.411 for men.

### Carotid ultrasonography

A single trained sonographer unaware of the study design scanned the carotid arteries using a Voluson 730 Expert B-mode ultrasonogram equipped with a 10-MHz probe (GE Healthcare). The right and left common carotid arteries were scanned from the proximal to the distal position, to the point of bifurcation. At the far wall of the right and left common carotid arteries, approximately 1 cm proximal to the carotid bulb, C-IMT was shown by two parallel lines that delineate the leading edges of the lumen-intima and media-adventitia interfaces. The mean value of the maximal thickness of each carotid artery was calculated to determine the C-IMT, as previously described^32^. C-IMT values above the 75th percentile cutoff point for the study population (≥0.65 mm) were considered increased^33^.

### Cardiovascular risk factors

The Framingham risk score was recommended by Adult Treatment Panel III as a standard risk assessment of CVD risk^34^. The indexes (gender, age, systolic blood pressure [SBP], LDL-c, HDL-c, and smoking status) used to generate the Framingham risk score were considered as the cardiovascular risk factors in the present study^34^.

### Statistical analysis

All statistical analyses were carried out with the SPSS 16.0 statistical software package (SPSS Inc., Chicago, IL, USA). The normality of data distribution was determined by the one-sample Kolmogorov-Smirnov test. Continuous variables are expressed as mean ± standard deviation or median with interquartile range. Categorical variables are expressed as number with percent. Comparisons between the two groups were conducted with the unpaired Student’s t-test, Mann-Whitney U-test and Chi-squared test for continuous data with a normal distribution, continuous data with a skewed distribution, and categorical variables, respectively. Multivariate logistic regression models were established to analyze the associations of a first-degree FHD with serum FGF23 levels and the OR of an increased C-IMT across serum FGF23 quartiles. All reported *P* values were two-tailed, and *P* < 0.05 was considered statistically significant.

## Additional Information

**How to cite this article**: Hu, X. *et al.* Elevation in fibroblast growth factor 23 and its value for identifying subclinical atherosclerosis in first-degree relatives of patients with diabetes. *Sci. Rep.* **6**, 34696; doi: 10.1038/srep34696 (2016).

## Figures and Tables

**Table 1:** Characteristics of the study participants.

Variable	Total	Without an FHD	With a first-degree FHD
Gender (men/women)	596/1123	507/900	89/223^*^
Age (years)	52.82 (46.25–57.73)	53.08 (45.72–57.93)	52.26 (47.61–57.02)
BMI (kg/m^2^)	22.89 (20.94–24.89)	22.89 (20.95–24.87)	22.91 (20.86–25.07)
W (cm)	79.00 (73.00–86.00)	79.00 (73.00–86.00)	79.00 (72.13–85.88)
SBP (mmHg)	120.00 (110.00–128.67)	120.00 (110.00–129.33)	120.00 (110.00–128.50)
DBP (mmHg)	76.67 (70.00–80.67)	76.67 (70.00–80.67)	76.67 (70.00–80.67)
FPG (mmol/L)	5.13 ± 0.41	5.12 ± 0.41	5.18 ± 0.41^*^
2 hPG (mmol/L)	6.00 (5.21–6.79)	5.99 (5.17–6.76)	6.07 (5.28–6.88)
HbA1c (%)	5.5 (5.3–5.7)	5.5 (5.3–5.7)	5.6 (5.4–5.8)^†^
FINS (mU/L)	6.55 (4.75–9.17)	6.46 (4.72–8.96)	7.15 (4.90–9.77)^*^
HOMA-IR	1.49 (1.05–2.11)	1.47 (1.04–2.09)	1.67 (1.12–2.32)^†^
TC (mmol/L)	5.04 (4.43–5.74)	5.02 (4.42–5.69)	5.17 (4.52–5.89)^†^
TG (mmol/L)	1.16 (0.81–1.63)	1.14 (0.80–1.63)	1.22 (0.86–1.67)
HDL-c (mmol/L)	1.45 (1.24–1.70)	1.46 (1.24–1.71)	1.42 (1.22–1.68)
LDL-c (mmol/L)	3.08 (2.57–3.62)	3.05 (2.52–3.57)	3.22 (2.69–3.74)^†^
CRP (mg/L)	0.56 (0.28–1.17)	0.56 (0.27–1.14)	0.58 (0.28–1.24)
Ca (mmol/L)	2.33 (2.27–2.40)	2.33 (2.27–2.40)	2.34 (2.27–2.40)
eGFR (mL/min/1.73 m^2^)	99.19 ± 11.88	99.04 ± 12.10	99.86 ± 10.84
C-IMT (mm)	0.58 ± 0.10	0.58 ± 0.10	0.58 ± 0.10
Current smoker, n (%)	339 (19.72)	277 (19.69)	62 (19.87)

Abbreviation: FHD: family history of diabetes; BMI: body mass index; W: waist circumference; SBP: systolic blood pressure; DBP: diastolic blood pressure; FPG: fasting plasma glucose; 2 hPG: 2-hour plasma glucose; HbA1c: glycated hemoglobin A1c; FINS: fasting serum insulin; HOMA-IR: homeostasis model assessment-insulin resistance; TC: total cholesterol; TG: triglyceride; HDL-c: high density lipoprotein cholesterol; LDL-c: low density lipoprotein cholesterol; CRP: C-reactive protein; Ca: Serum calcium; eGFR: estimated glomerular filtration rate. Data are means ± SD, median (interquartile range) or N (%).

*P < 0.05 versus subjects without an FHD; ^†^*P* < 0.01 versus subjects without an FHD.

**Table 2:** Association between the presence of a first degree FHD and serum FGF23 levels.

	Serum FGF23 levels		
	OR	95% CI	*P*
Unadjusted	1.023	1.009–1.037	0.001
Adjusted for age, gender and eGFR	1.025	1.010–1.039	0.001
Adjusted for C-IMT	1.024	1.010–1.038	0.001
Adjusted for cardiovascular factors	1.022	1.007–1.036	0.003

Cardiovascular factors include age, gender, SBP, LDL-c, HDL-c, and smoking status.

**Table 3:** Logistic regression analysis of the presence of an increased C-IMT.

Serum FGF23 levels	Without an FHD			With a first-degree FHD		
	OR	95% CI	*P*	OR	95% CI	*P*
Per unit	1.016	1.002–1.030	0.026	1.030	1.000–1.060	0.047
An increased C-IMT						
Quartile 1	Reference			Reference		
Quartile 2	1.119	0.790–1.587	0.526	1.563	0.661–3.693	0.309
Quartile 3	1.415	1.011–1.980	0.043	1.527	0.633–3.685	0.347
Quartile 4	1.439	1.033–2.003	0.031	2.263	1.010–5.069	0.047

Serum FGF23 levels: Quartile 1: ≤ 23.80 pg/mL; Quartile 2: 23.81–29.19 pg/mL; Quartile 2: 29.20–34.99 pg/mL; Quartile 4: ≥ 35.00 pg/mL.
